# Hold the salt for kidney regeneration

**DOI:** 10.1172/JCI181397

**Published:** 2024-06-03

**Authors:** Yun Xia, Thomas M. Coffman

**Affiliations:** 1 Lee Kong Chian School of Medicine, Nanyang Technological University, Singapore.; 2 Cardiovascular and Metabolic Disorders Signature Research Program, Duke-NUS Medical School, Singapore.

## Abstract

The macula densa (MD) is a distinct cluster of approximately 20 specialized kidney epithelial cells that constitute a key component of the juxtaglomerular apparatus. Unlike other renal tubular epithelial cell populations with functions relating to reclamation or secretion of electrolytes and solutes, the MD acts as a cell sensor, exerting homeostatic actions in response to sodium and chloride changes within the tubular fluid. Electrolyte flux through apical sodium transporters in MD cells triggers release of paracrine mediators, affecting blood pressure and glomerular hemodynamics. In this issue of the *JCI*, Gyarmati and authors explored a program of MD that resulted in activation of regeneration pathways. Notably, regeneration was triggered by feeding mice a low-salt diet. Furthermore, the MD cells showed neuron-like properties that may contribute to their regulation of glomerular structure and function. These findings suggest that dietary sodium restriction and/or targeting MD signaling might attenuate glomerular injury.

## Functions of MD to coordinate tissue regeneration

The macula densa (MD) has a well-established role in controlling secretion of renin, the rate-limiting step in activation of the renin-angiotensin system (1). Low levels of sodium and chloride in distal tubular fluid are sensed by MD cells, which initiate cyclooxygenase-2–dependent (COX-2–dependent) production of prostaglandin E2 (PGE_2_), triggering the release of renin from juxtaglomerular (JG) cells (2). In this issue of the *JCI*, Gyarmati and colleagues leveraged intravital multiphoton microscopy and genetic fate tracing in mice to clearly demonstrate MD cell–dependent activation of dynamic tissue remodeling in the glomerular pole by low-salt feeding (3). The MD recruited endothelial and mesenchymal progenitors, which gave rise to vascular, interstitial, and epithelial cells (3). This tissue remodeling process was dependent on well-established MD signaling pathways via COX-2 and neuronal nitric oxide synthase (4). Furthermore, MD cells activated by low levels of dietary salt expressed a range of tissue angiogenic and remodeling genes, including *Ccn1* and *Ccn3*, which have not been extensively investigated (3). Finally, this program depended on Wnt signaling, consistent with the well-established linkage of Wnt pathways to remodeling and repair in other systems (5).

## Neuronal-like MD cells as possible intercellular crosstalk centers

**Figure 1:**
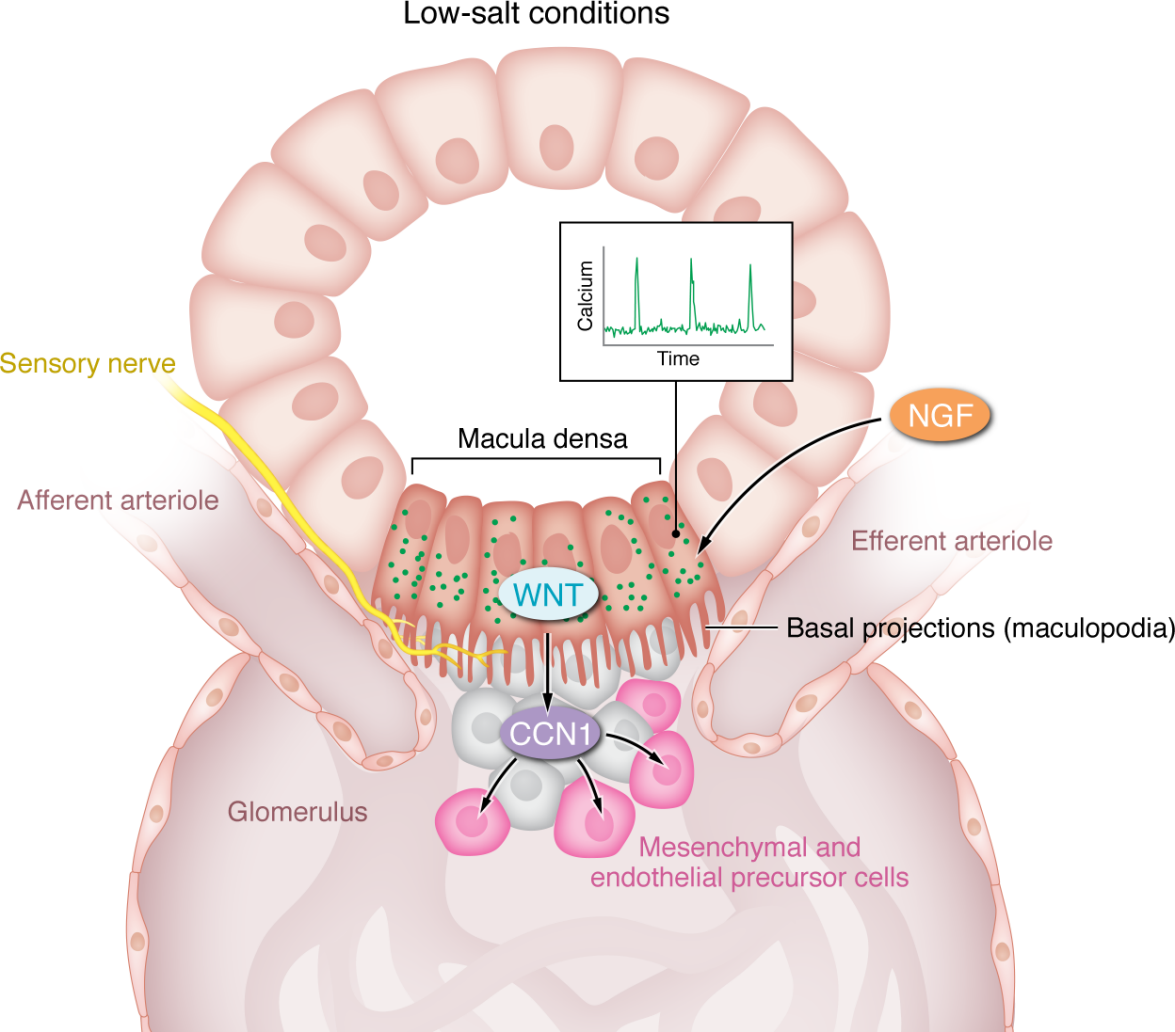
Cells in the MD have neuronal-like properties and promote transformation of progenitor cells. In the juxtaglomerular apparatus, MD cells form axon-like cell projections called maculopodia from the basal surface. Calcium oscillations within these cells synchronize with rhythmic changes in the diameter of adjacent glomerular arterioles. MD cells also released CCN1 to recruit endothelial and mesenchymal progenitors. Notably, low-salt conditions promote CCN1 release and may reduce glomerular pathology via Wnt/β-catenin and NGF/NGFR signaling.

As discussed above, the classic homeostatic functions of MD cells are effected through release of locally acting mediators such as prostanoids or adenosine (6, 7). In their study, Gyarmati and colleagues provided compelling evidence that MD cells exhibit characteristics resembling those of neurons, raising the possibility that direct cell-to-cell communications influence glomerular physiology and structure. For example, using advanced imaging techniques, this group had previously shown that low-salt feeding induces formation of axon-like cell projections from the basal surface of MD cells, which they termed maculopodia (Figure 1) (8). In Gyarmati et al., intravital imaging of intracellular Ca^2+^ revealed robust Ca^2+^ transients in MD cells (3). This autonomous Ca2^+^ signaling activity was spatially confined to the MD plaque without propagation to adjacent tubular or vascular cells (3). However, calcium oscillations were synchronized with rhythmic changes in the diameter of adjacent glomerular arterioles, suggesting a possible functional association (3).

To further explore the molecular basis of MD responses, a unique set of single-cell and bulk transcriptomic profiles of MD and adjacent tubular epithelial cells were generated from mice on standard and low-salt diets. Robust expression of several neuronal genes was found in MD cells, including nerve growth factor receptor (*Ngfr*), tyrosine hydroxylase, and synaptophysin (3, 9). A deeper mining of a single-cell data set demonstrated enrichment of pathways associated with neuronal biological processes, such as axon guidance, synaptic functionality, vesicle exocytosis, and membrane depolarization (3). The findings of close anatomical association between MD cell basal processes and the sympathetic and sensory nerve endings (Figure 1), along with the high expression of synaptic transmission genes, raises the possibility of interaction and/or communication between MD cells and the sympathetic nervous system. Indeed, previous studies have suggested that sympathetic innervation may modulate regenerative responses in other tissues (10, 11).

## MD cells and kidney tissue regeneration

The mammalian kidney has a limited capacity to regenerate, likely due to the exhaustion of nephron progenitors during development (12). Nevertheless, harnessing regeneration and repair pathways to reverse kidney injury has been a holy grail in adult mammalian kidney (13, 14). While previous studies have demonstrated the propensity for low-salt diet and renin-angiotensin system inhibition to stimulate remodeling of the JG apparatus vasculature (15), Gyarmati and colleagues extended this work, showing dynamic induction of progenitor cells during low-salt feeding with differentiation into multiple cell lineages (3). Furthermore, they showed that MD cell–mediated tissue regeneration was dependent on Wnt/β-catenin signaling, a key regulator of kidney embryogenesis (16). While Wnt/β-catenin signaling is usually suppressed in adult kidney, reactivation occurs during injury repair and regeneration (17). Notably, compared with other kidney cells, MD cells have high levels of WNT at baseline that are further increased by low-salt feeding (3).

Repair and regeneration of injured tubular epithelial cells has been extensively studied, but comparatively little is known about whether and how endogenous kidney mesenchymal cells contribute to tissue repair and regeneration after injury. Genetic lineage tracing has demonstrated multilineage potential of renin cells in glomerular injury (18), giving rise to podocytes, parietal epithelial cells, and mesangial cells (19, 20). Whether MD cells play a role to induce proliferation and differentiation of multipotent renin cells or trigger direct lineage conversion (termed transdifferentiation) of renin cells to other renal cell types is an interesting area for future research (21).

## MD generated factors for glomerular protection

Low sodium in tubular fluid triggers MD cells to release angiogenic and growth factors such as CCN1. Gyarmati and colleagues tested the capacity of these factors to attenuate glomerular disease in a mouse model of adriamycin-associated glomerulopathy. Administration of pharmacological concentrations of CCN1, or supernatants from MD cells that had been incubated with low-sodium media, reduced albuminuria and attenuated the severity of glomerular pathology (3). In normal human kidney, immunolabeling enabled detection of a prominent CCN1 signal, consistent with robust expression observed in the RNA expression studies (3). By contrast, CCN1 content in MD cells and global kidney expression of CCN1 were reduced in patients with CKD, and there was an association between low urinary CCN1 and reduced eGFR, suggesting a possible causal association.

An evolutionarily conserved function of the kidney is to protect against sodium and water loss in order to maintain body fluid balance. The MD plays a critical role in these responses. Accordingly, downstream actions of the MD extending to areas of regeneration and repair are consistent with its role in the regulation and support of homeostasis. Chronic low-salt feeding was sufficient to induce the regenerative and angiogenic program, but these effects were augmented by concomitant blockade of the renin-angiotensin system with an angiotensin-converting enzyme inhibitor (ACEi). As human MD cells do not express angiotensin receptors (22), augmentation by ACEi may be secondary to other physiological consequences of ACE inhibition, such as blood pressure lowering, or inhibition of Ang II–dependent modulation of target cells in the glomerulus. Similar synergistic effects of combining low-salt diet and renin-angiotensin system blockade have been observed on JG apparatus remodeling responses (15). In the latter case, reduced blood pressure activates baroreceptor mechanisms, providing complementary stimulation of renin and JG cell recruitment (23).

## Moving toward clinical applications

One of the interesting questions raised by Gyarmati and colleagues (3) is whether MD signaling could be systematically harnessed to promote kidney protection. Because every glomerulus is paired with a MD, the potential for coordinated activation of beneficial MD signals to influence glomerular health could be quite dramatic, and there is precedent for this. For example, MD signaling may play a crucial role in the efficacy of SGLT2 inhibitors, which substantially reduce the risk of kidney disease progression in both diabetic and nondiabetic kidney disease (24, 25). SGLT2 inhibitors specifically inhibit glucose reabsorption in the proximal tubule, resulting in enhanced delivery of solutes to the distal nephron (26), triggering MD-dependent release of vasoconstrictors, such as adenosine, resulting in vasoconstriction of afferent glomerular arterioles, reducing glomerular filtration rate and lowering of injurious glomerular pressures (27).

Based on the observations of Gyarmati and colleagues (3), a simple intervention, such as dietary sodium restriction, perhaps in combination with renin-angiotensin system blockade, might promote a favorable profile of MD signaling that would enhance glomerular health. In this regard, clinical studies have demonstrated effects of low-salt diet to reduce proteinuria in patients with CKD, and the reduction of proteinuria observed with low-salt diet plus ACEi was greater than that of either intervention alone (28). It remains to be determined whether these effects are a consequence of MD-related release of regenerative factors and/or whether exogenous administration of critical factors such as CCN1 might be effective clinical renoprotective therapies. Furthermore, it should be noted that dysregulated production of angiogenic mediators can promote glomerular injury (29), while aberrant upregulation of Wnt/β-catenin may lead to fibrosis and podocyte damage (17). In future studies, potential adverse effects from chronic activation of these regenerative and angiogenic signaling pathways must also be considered.

## Version 1 06/03/2024

Electronic publication
